# Conjugation of TLR7 Agonist Combined with Demethylation Treatment Improves Whole-Cell Tumor Vaccine Potency in Acute Myeloid Leukemia

**DOI:** 10.7150/ijms.49983

**Published:** 2020-08-27

**Authors:** Guocheng Zhong, Guangyi Jin, Wei Zeng, Changhua Yu, Yan Li, Ji Zhou, Li Zhang, Li Yu

**Affiliations:** 1 Department of Hematology and BMT center, the first Medical Center, Chinese PLA General Hospital. Beijing 100853, China.; 2 Department of Hematology and Oncology, Shenzhen University General Hospital, Shenzhen 518055, China.; 3 Carson International Cancer Center, Department of Medicine, Shenzhen University, Shenzhen 518037, China.

**Keywords:** AML, TLR7, SZU-106, vaccine.

## Abstract

***Background:*** Acute myeloid leukemia (AML) is a malignant hematological disease with high refractory rate. Immune escape of AML cells is one of the underlying mechanisms mediating the relapse of the cancers. Various immunotherapies based on the 'patients' immune response to tumor cells have been developed to targeting the immune escape of AML cells, which lead to the minimal residual disease (MRD) after treatment. But the efficacy of those treatments or the combination of treatments remains unsatisfactory.

***Methods:*** A Toll-like receptor (TLR)-7 agonist SZU-106 was chemically synthesized. SZU-106 was conjugated to Decitabine (DAC), a demethylation agent, treated AML cells to construct tumor vaccine. The potency of the newly constructed AML cell vaccine, SZU-106-DAC-AML was tested *in vitro* and *in vivo* for the expression of tumor antigens and the activation level of immune responses.

***Results:*** Compared to the well characterized TLR7 agonist R848, SZU-106 has a comparable potency to activate TLR7 signaling pathway. SZU-106-DAC-AML, constructed by conjugating SZU-106 to DAC treated tumor cells, exhibited increased expression of tumor antigens, and enhanced the activation of DC cells and T cells *in vitro* and *in vivo*. The result of xenograft tumor model showed that SZU-106-DAC-AML tumor vaccine greatly inhibited tumor growth and prolonged animal survival.

***Conclusions:*** Conjugation of TLR7 agonist combined with up-regulation of tumor antigen expression improved the effectiveness of whole-cell tumor vaccine in AML.

## Introduction

Acute myeloid leukemia (AML) is a group of malignant hematological diseases that are phenotypically and genetically heterogeneous 1. It is the most common type of acute leukemia and brings a substantial economic burden due to its high mortality rate and the associated high medical cost 2-4. The incidence rate of AML is about 2.25/100,000, and over 10000 cases per year in the United States alone, representing 80-90% of the adult acute leukemia 4-7. Although chemotherapy combined with stem cell transplantation has been proved effective to treat AML, the most common cause of death is bone marrow failure, 8 only 30-40% of young patients and less than 20% of old patients could achieve long-term, disease-free survival 9, 10. Therefore, it remains imperative to develop new therapeutic methods to treat AML.

Minimal residue disease (MRD), a small reservoir of treatment-resistant leukemic cells that persist after chemotherapy, is an important risk factor for the poor prognosis of AML and often accounts for the relapse of the disease 11. Allogeneic hematopoietic stem cell transplantation (HSCT) can be used to clear MRD effectively and has a positive impact on relapse rate and survival 12. However, HSCT is still associated with relatively high morbidity and mortality rate, resulting in the limitation of its application in relatively younger patients who generally have fewer co-morbidities 13.

In AML, leukemic cells could evade the host immune system from various respects, such as decreasing antigenicity of AML cells, downregulation of non-self human leukocyte antigen, and down-regulation of host dendritic cells (DC) activity and T cell activity 14-18. Recently, immunotherapy, as a therapeutic strategy to treat AML, has been more and more studied 19. Different immunotherapy methods have been developed and proved effective to certain extents. Some examples of these methods are monoclonal antibodies (MoAbs) and immuno-chemotherapy, vaccinations against AML antigens, and chimeric antigen receptor T-cells (CAR T-cells) 20. The rationale underlying these methods to increase the immunogenicity of tumor cells, such as tumor cell vaccination 21, or to improve the functionality of DC cells or T cells, such as dendritic cell-T cell (DC-T) adoptive immunotherapy 22, 23.

Allogeneic or autologous tumor vaccines could potentially work as immunotherapy enhancing agents 21. It can be prepared by irradiating whole tumor cells or cell lysates, with or without modifications 20. The purpose of a tumor vaccine is to enhance tumor antigen-specific immunogenicity. Decitabine (DAC), also known as 4-Amino-1-(2-deoxy-beta-D-erythro-pentofuranosyl)-1,3,5-triazin-2(1H)-one, is an analogue of cytidine. It has been reported that low dose DAC could inhibit the activity of DNA methyltransferase (DNMT) from reversing the gene silencing caused by high methylation level of DNA in tumor cells 23. Administration of DAC up-regulates the expression of tumor-associated antigens and/or immune response associated molecules, and consequently, increases the immunogenicity of tumor cells 21.

Toll-like receptor 7 (TLR7), a member of the TLR family, is believed to be involved in the anti-virus innate immune response, antitumor processes, and the regulation of immune response 22. It has been found that TLR7 agonist (TLR7a) can be used in combination with chemotherapy, radiotherapy, and other immunotherapies to increase the immune response and to induce tumor cell apoptosis 24, 25. For example, a well-characterized TLR7 agonist R848, also called resiquimod, has been demonstrated that it works as an antitumor reagent in a mouse model of lymphoma when combined with radiation by priming antitumor immune response 26. Our lab has previously synthesized an effective TLR7 agonist, SZU-101^27^, ^28^, which also showed antitumor activity in a murine model of T cell lymphoma.

Based on these observations, we wonder if a treatment combining the enhancement of tumor antigen exposure and host immune response would demonstrate an improved antitumor effect. In the present study, we treated AML tumor cells with DAC, and conjugated a new TLR7 agonist SZU-106, which is derived from the previous synthesized drug SZU-101, to the DAC treated cells. Compared to SZU-106 alone or DAC treated AML tumor cells, the DAC treated tumor cells conjugated with SZU-106 demonstrated increased ability to induce DC cell maturation and T cell activation *in vitro* inhibited subcutaneous tumor growth in a Balb/c mice model, and improved tumor-bearing mice survival. The mentioned function suggesting that a combination of antigen exposure and immune response enhancement may be a promising strategy to improve the efficiency and specificity of dendritic cells-cytotoxic T lymphocytes (DC-CTL) based immunotherapy.

## Materials and Methods

### Mice and cell lines

The Balb/c mice used in this study were purchased from Guangdong Medical Laboratory Animal Center (Guangdong, China) and were maintained under pathogen-free conditions in the animal facility. All procedures involving mice were approved by the Institutional Animal Care and Use Committee of Chinese PLA General Hospital and General Hospital of Shenzhen University. All experiments were conducted in accordance with the US Department of Health and Human Services Guide for the Care and Use of Laboratory Animals and institutional guidelines. WEHI3 (mouse leukemia cell line), U937 (human myeloid leukaemia cell line), Raji (human B lymphoblastoid cell line), Z-138 (human B lymphoblastoid cell line), Hut-78 (cutaneous T cell lymphoma cell line), Jurkat (human T lymphocyte cell line), Molt-4 (human T lymphoblast cell line), Kasumi-1 (human acute myeloid leukemia cell line), NB-4 (acute promyelocytic leukemia cell line), THP-1 (human monocytic cell line), and K562 (human immortalized myelogenous leukemia cell line) cells were purchased from ATCC (Manassas, VA, USA) and cultured according to the guidelines provided by ATCC.

### Isolation and generation of human DC cells and T cells

20 ml peripheral blood was collected, and an equal amount of physiological saline was added to the blood sample and mixed well. Ficoll-Paque Plus medium (GE, #17144002, USA) was then carefully added into the sample followed by break-free centrifugation at 2000 rpm for 20 minutes at room temperature. After centrifugation, the white-membrane layer (mono-nuclear cells layer) was carefully removed to a culture dish containing RPMI-1640 medium (Thermo Scientific, USA) and cultured in an incubator for 2 hours at 37℃. Two hours later, the cell-containing culture medium was removed to a new dish for CTL isolation. The cells attached to the culture dish were washed with RPMI-1640 medium and further cultured in fresh medium with recombinant granulocyte-macrophage colony-stimulating factor (rhGM-CSF) and recombinant interleukin 4 (rhIL-4) (125ng/ml, Schering-Plough, Kenilworth, USA). rhGM-CSF and rhIL-4 were re-added to the culture medium on day 3 and day 5 at the same concentration. At day 6, tumor necrosis factor α (TNF-α) (2µg/ml, PeproTech, # 315-01A, USA) was added to the medium to promote the maturation of DC cells. CD83 (clone HB15e), human leukocyte antigen II (HLA-II, clone Tu39), and CD86 (clone BU63) (BioLegend, USA) were used to validate the purity and maturation of the generated DC cells.

The above-mentioned CTL containing medium was mixed well and the cells were counted and further cultured in IL-2 containing medium for 5 days. The CD8+ T cells were isolated by MojoSort™ Human CD8 T Cell Isolation Kit (BioLegend, #480011, USA). The percentage of CD8+ population over 90% was considered as acceptable for experiments.

### Isolation and generation of mouse DC and T cells

Mouse DC cells: Healthy Balb/c mouse was sacrificed by neck dislocation and soaked in 75% alcohol for 5 minutes. The mouse body was moved to a sterile Laminar hood and the back legs above the hip joint was cut out to access the femur and tibia. Muscle and tissues on the cut-out back legs were debrided with sterilized scissors and forceps, and the cleaned bones were then washed with PBS twice. Both ends of the bone were cut with sterilized scissors as close to the joints as possible, the needle of a syringe filled with FACS solution (PBS containing 2% fetal bovine serum) was inserted into the bone to flush the bone marrow out until the bone turned completely white. The bone marrow was then repeatedly pipetted and resuspended in FACS and filted with 200 guage filter to remove muscles, tissues and bone debris. Resulted cell suspension was removed to a 15ml centrifuge tube, centrifuge at 1400 rpm for 5 minutes and the supernatant was discarded. 1ml ACK lysis buffer (Beyotime Biotechnology, #3702, Shanghai, China) was added to the tube to resuspend the cells for 1 minute. 5ml RPMI-1640 medium was added into the tube to stop the reaction and cells were re-centrifuged for 5 minutes at 1400 rpm, discarded the supernatant. 10 ml RPMI-1640 complete medium was used to resuspend the cells and the cells were then cultured in a 100mm culture dish for 6 hours. The attached cells were then resuspended by pipetting, centrifuged, resuspended in X-VIVO medium (Lonza, #04-418, Guangzhou, China) containing 10% fetal bovine serum and counted. Then cells were seeded in 24 well plate at the density of 1 million cells per well. GM-CSF and IL-4 were added to the culture medium at the concentration of 20ng/ml and 10ng/ml, respectively. This is considered as day 0 of culturing. At day 3 and day 5, cell medium was half-refreshed and the GM-CSF and IL-4 was added to the medium accordingly to achieve the original concentration.

Mouse CTL from splenocytes: Balb/c mouse was sacrificed, and the spleen was dissected, cut into pieces, grinded in 4ml Ficoll-Paque Plus medium, and filtered with 0.22 µm filter. The filtered cell suspension was then pipeted and removed to a 15ml tube. 1ml RPMI-1640 medium was added into the tube slowly along the tube wall to cover the cell suspension, then centrifuged at 800g for 30 minutes. After centrifugation, the white layer in the tube (lymphocytes layer) was removed to a new 15ml tube and 10ml RPMI-1640 medium was added and mixed well. The mixture was then centrifuged at 250g for 10 minutes, the supernatant was discarded. 1ml ACK lysisbuffer was used to resuspend the cells for 1 minutes followed by adding 4ml complete RPMI-1640 medium, centrifuging at 1000 rpm for 5 minutes and discarding the supernatant. The cell pellet was then resupended in 2ml complete RPMI-1640 medium, counted and cultured in medium containing IL-2 for 5 days.

### DAC treatment of AML cells

Decitabine (Dacogen^®^, Xian-Janssen pharmaceuticals Ltd, China) was dissolved in phosphate-buffered saline (PBS) (pH 7.4) to obtain 100 µM stocks and stored at -20℃. Mouse leukemia cell line WEHI3 was seeded in 96 well plated at 1x10^5^ /well and was treated with different concentrations of DAC for 3 days. PBS was used as control. CCK8 cell viability detection kit (Dojindo, #CK04, China) was used to determine the IC50 of DAC.

After determination of the IC50 of DAC, indicated leukemia cell lines were treated with 1 µM DAC for 3 days, and the expression of tumor antigens was detected by reverse transcription- PCR.

### Reverse Transcription-PCR (RT-PCR)

Total RNA was extracted from DAC-treated or untreated cells using Trizol reagent (Thermo Scientific, #15596026, USA) according to the 'manufacturer's instruction. RT was performed using the Reverse Transcription System (Promega, #A3500, USA) on 1 mg of total RNA, and PCR amplifications were then performed using primers shown in **Table 1** 26**.** Simultaneous amplification of β-actin was used as an internal control for the amount and integrity of the RNA analyzed. All PCR products were resolved on agarose gels and visualized using ethidium bromide staining.

### Synthesis of SZU-106

**Figure 1:**
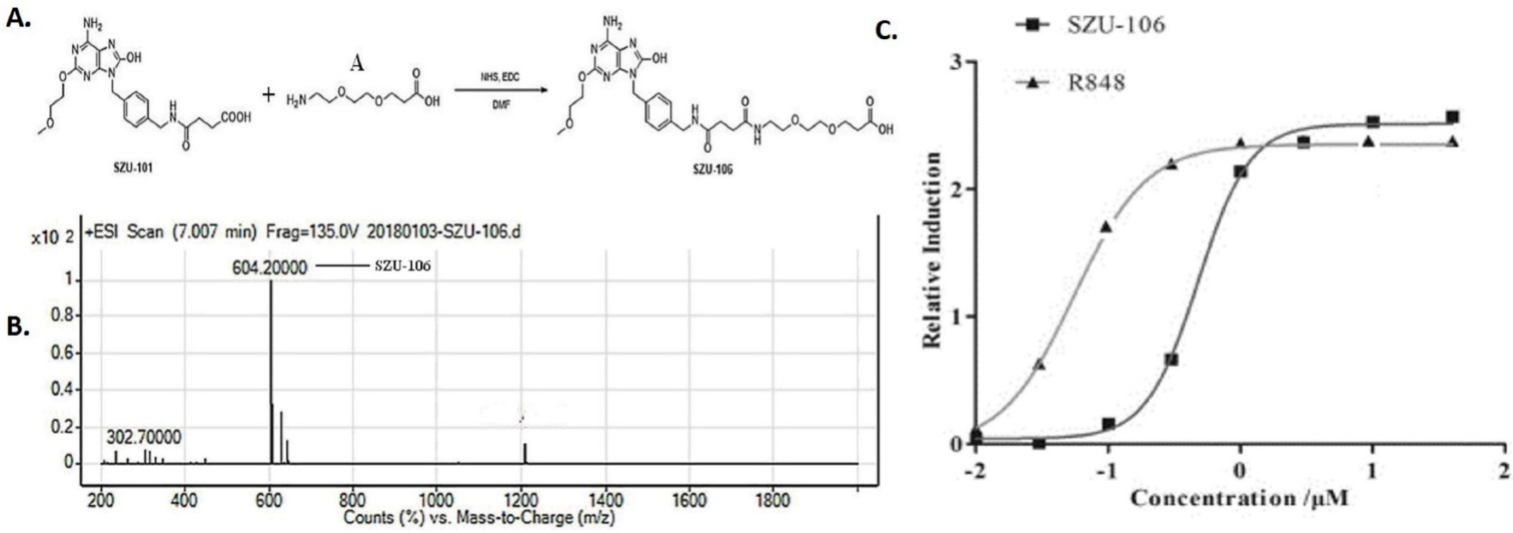
** Synthesis of SZU-106. A.** diagram showing the procedure to synthesize SZU-106 from SZU-101(our previous product). **B.** The molecular weight of SZU-106 was 604.2 g/mol illustrated by LC-MS. **C.** TLR7 reporter assay in HEK-BLUETM hTLR7 cells. Indicated concentrations of SZU-106 was used to treat hTLR7 cells and the reporter gene expression was detected to determine the activation of TLR7 signaling pathway. R848 was used as positive control. Each concentration was triplicated, and the graph was calculated from the result of three independent repeat of experiments.

SZU-101 is required for the synthesis of SZU-106, after following our established SZU-101 synthesis protocol, the essential compound 101-1 and 101-2 were mixed and stirred at RT for 12 h and heated at 60°C for 1 h. The reaction mixture was concentrated and then dissolved in 10% HCl to obtain compound SZU-101 28-31, the equivalent amount of synthesized SZU-101 and compound A (**Figure 1A**) were added into a flask, mixed, and reacted in the presence of active ester and DMF at room temperature overnight. The synthesized product was collected and named SZU-106. Compared to SZU-101, SZU-106 has one more carboxyl group, which allows SZU-106 to be conjugated to cells. Synthesized product was further purified with a silica gel column with a mixture of CH_2_Cl_2_ and CH_3_OH at the ratio of 1:1. The analytical quantity and the purity of the purified product were analyzed by LC-MS (liquid chromatography-mass spectrometer).

### HEK-BLUE assay

SZU-106 was synthesized as described above. HEK-BLUE hTLR7 cells were purchased from InvivoGen (San Diego, CA). This cell line stably expresses human TLR7 and a SEAP reporter, which can be used to detect TLR7 agonism through the activation of NF-kB signaling. The cells were maintained in selective DMEM growth medium with an additional 10 μg/ml blasticidin and 100 μg/ml Zeocin™ (Fisher Scientific, #R25001, USA). After incubation with different doses of SZU-106 for 2 days, the reporter gene SEAP expression was detected using the HEKBLUE detection kit according to the 'manufacturer's instruction. The TLR7 agonist R848 was used as the positive control.

### Construction of SZU-106-DAC-AML tumor vaccine

Purified SZU-106 was firstly esterified in the presence of DMSO for 6 hours at room temperature. Then esterified SZU-106 was conjugated to indicate tumor cells. The chemical reactions were illustrated below. In detail, AML cells were treated with 1µM DAC for 3 days, trypsinized, centrifuged, and counted. One cell could be conjugated with 1x10^9^ SZU-106 molecules. The amount of needed esterified SZU-106 was calculated based on the cell number. The required amount of esterified SZU-106 was added into cell cultures, and the mixture was rotated on a shaker at 1400 rpm for 1 hour at room temperature for conjugation. After conjugation, the mixture was centrifuged, and the supernatant was discarded. The SZU-106 conjugated DAC treated cells were washed twice and resuspended in PBS. The cell number was counted. Then the cells were sterilized by ultraviolet irradiation. Two cell lines were used to construct SZU-106-DAC-AML tumor vaccines: mouse leukemia cell WEHI3 and human leukemia cell line U937 followed the method described above.

### SZU-106-DAC-AML tumor vaccine treatment of human DC and DC-CTL cells *in vitro*

U937 cells were treated with DAC and conjugated with SZU-106, as described above. DAC-treated only U937 cell vaccine and SZU-106 conjugated, DAC untreated U937 cell vaccine were also constructed as above. The resulted tumor vaccines were then mixed with DC cells or DC-CTL cells, both at 1:10 ratio. 24 hours later, indicated factors were measured.

### Detection of indicators for immune cells by flow cytometry

The immune phenotypes of DC and CTL cells were identified and analyzed by flow cytometry using BD FACSCalibur (BD Biosciences, USA). CD25, CD69, CD80, CD86, HLA-II (clone BC96, FN50, 2D10, BU63, Tu39, respectively, BioLegend, USA) were used to evaluate the immune phenotypes of isolated DC cells and/ or splenocytes. The flow cytometry data were analyzed by FlowJo V10 (FlowJo, USA).

### ELISA

DC cells or DC-CTL cells were stimulated with indicated tumor vaccines ELISA kits detecting mouse IL-6 and IFN-γ (BioLegend, USA) were used to evaluate the cytokine secretion of isolated mouse splenocytes upon SZU-106 stimulation following 'manufacturer's instruction. ELISA kits detecting human IL-6, IFN-γ, and TNF-α (BioLegend, USA) were used to identify the cytokine secretion of isolated human T cells upon indicated tumor vaccine stimulation.

### Cell viability

WEHI3 cells were seeded in 96 cell plates at 1x10^5^ cell/well overnight. DAC was added into the culture medium at 0.1, 1, 10, 100 µM for 24 hours. Then CCK-8 (Dojindo Molecular Technologies, Rockville, USA) kit was used to measure the cell viability following the 'manufacture's instruction. Briefly, 10 ul CCK-8 solution was added to each well and incubated for 2 hours in the cell culture hood. 2 hours later, the absorbance of 450 nm was measured by a microplate reader. For each concentration of DAC was tested in 5 wells.

### Tumorigenesis and tumor vaccine treatment *in vivo*

5x10^4^ U937 cells were injected in 4-6 week-old Balb/c mice subcutaneously. Treatment was started when the diameter of tumors reached 5 mm. Mice bearing tumors were randomly divided into 4 groups (n=20), and the indicated sterilized tumor vaccines were subcutaneously injected surrounding the tumor once a week. Tumor size was measured every 2 days, and the mice's survival was recorded as well until the end of the experiments. The following formula was used to calculate the tumor volume. Volume = (1/2 longest diameter) x (shortest diameter)^2^

### Statistics

To determine whether statistically significant differences exist among groups, clinical scores or tumor volume differences were analyzed by using ANOVA test. For comparison of mouse survival, the Kaplan-Meier survival analysis and log-rank test were used. Other results were compared by using the Student t-test. A *p* < 0.05 was considered statistically significant.

## Results

### Synthesis and functional characterization of SZU-106

**Figure 2:**
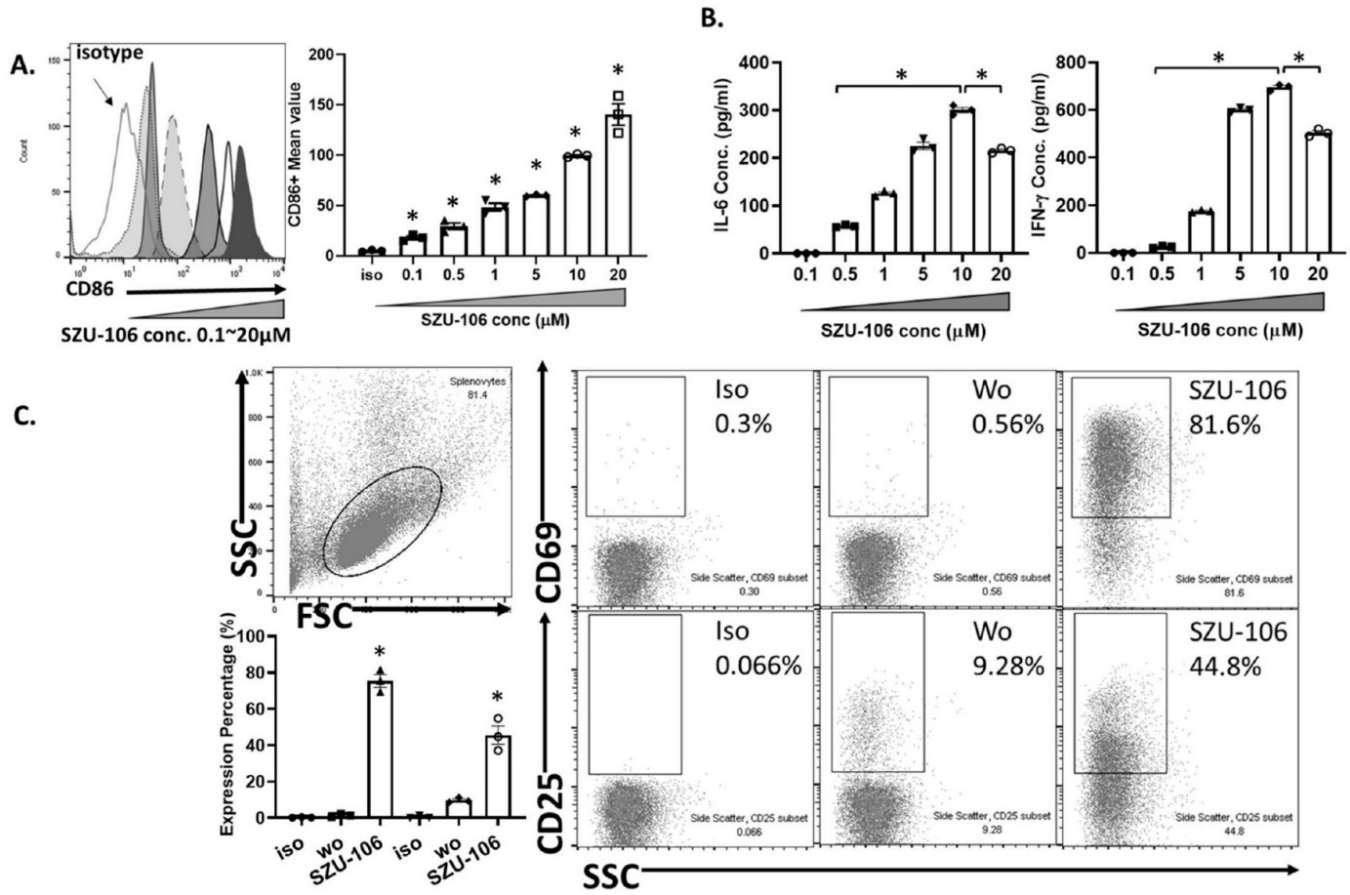
** Synthesized SZU-106 effectively activated TLR7 signaling pathway in DC and splenocytes. A.** SZU-106 stimulation of isolated mouse DC cells. Stimulated DC cells was measured for the CD86 expression by flow cytometry. The experiment was done three times independently and the result was presented as mean ± SEM, statistically significant differences were calculated by one-way ANOVA test, *p < 0.05. **B.** SZU-106 stimulation of mouse splenocytes. Various concentration of SZU-106 was used to treat isolated mouse splenocytes and the concentration of indicated cytokines in the culture medium was measured by ELISA. Cells were treated for 24 hours as described in the Method section**.** The experiment was done three times independently and the result was presented as mean ± SEM. n=3, statistically significant differences were calculated by one-way ANOVA test, *p < 0.05. **C.** CD25 and CD69 expressions in mouse splenocytes were measured 24 hours after SZU-106 treatment by flow cytometry.

In our previous study, a TLR7 agonist SZU-101 was successfully synthesized 27. However, SZU-101 ' could not be conjugated to intact cells due to the lack of carboxyl groups in its structure. In order to make it possible to conjugate the TLR7 agonist to tumor cells, we added a carboxyl group to SZU-101 and synthesized a new chemical SZU-106. The molecular weight of SZU-106 was 604.2 g/mol, as analyzed by LC-MS and molecular formula shown in **Figure 1B**. A reporter assay was used to test if the modified TLR7 agonist SZU-106 could activate TLR7 signaling pathway. As shown in **Figure 1C**, HEK-BLUE^TM^ hTLR7 cells, which was designed to monitor the activation of TLR7 signaling pathway via the activation of NK-κB, was stimulated with SZU-106 at various concentrations. R848 (Enzo Life Sciences, USA), a well-documented TLR7 agonist, was used as a positive control. The result demonstrated that along with the increase of SZU-106 concentration, the intensity of the report gene induction increased. When the concentration of SZU-106 reached 10 µM, it demonstrated a comparable ability to activate TLR7 signaling pathway as R848. We then tested if SZU-106 could activate the immune responses in mouse DC cells and splenocytes. As shown in **Figure 2A**, Mouse DC cells were activated by SZU-106 stimulation in a dose-dependent manner, as suggested by the increased percentage of CD86+ cells. Isolated mouse splenocytes were also used to test the efficacy of SZU-106. SZU-106 was added into the culture medium at indicated concentrations for 24 hours, and the levels of IL-6 and IFN-γ in the culture media were measured by ELISA **(Figure 2B)**. The result showed that the secretion of IL-6 and IFN-γ reached the highest level when the concentration of SZU-106 was 10 µM. When the concentration of SZU-106 reached 20 µM, the cytokine production was inhibited. In order to check the status of T cell activation, the expression level of CD25, CD69 on the cell surface was analyzed by flow cytometry in untreated or SZU-106 treated cells. The result was shown in **Figure 2C.** CD25+ and CD69+ T cell population significantly increased (from 9.28% to 44.8%, and 0.56% to 81.6%, respectively) after SZU-106 treatment, suggesting that SZU-106 has a good potency to activate T cells.

### DAC treatment increased the expression of tumor antigens expression in AML cells

**Figure 3:**
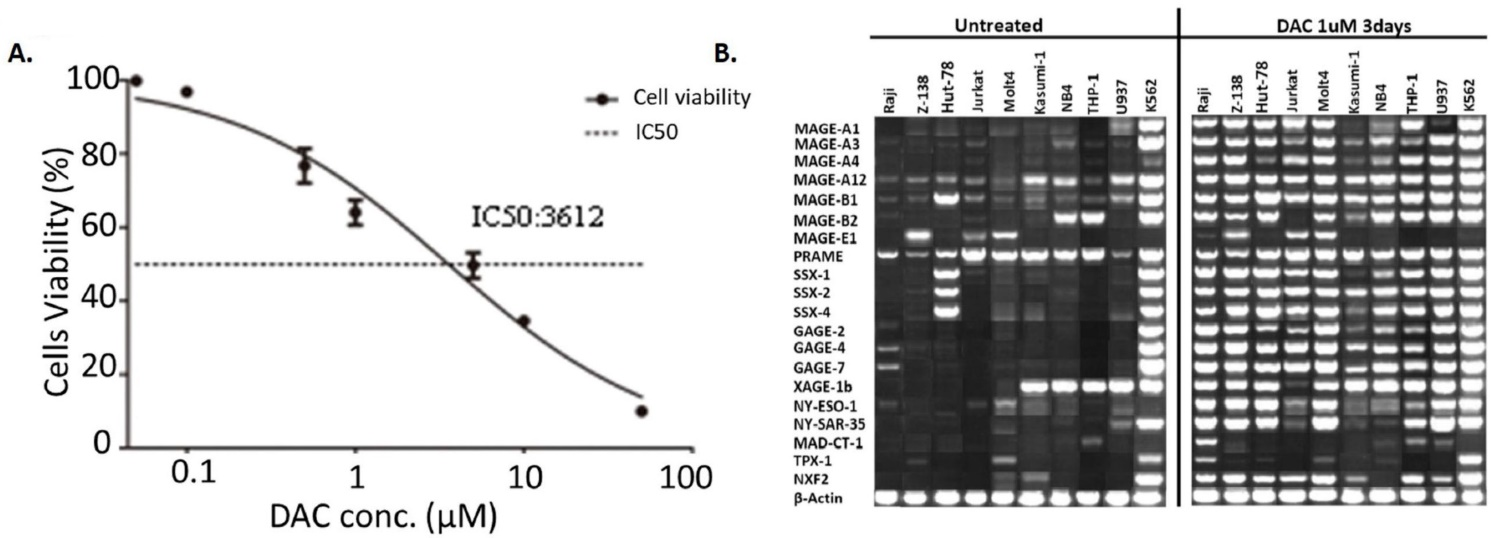
** Expression of tumor antigens increased upon DAC treatment in AML cells. A.** IC50 determination of DAC in mouse WEHI3 cells. Cells were treated with indicated concentration of DAC and cell viability was measured by CCK8 kit. Each concentration was triplicated, and the result was calculated from 3 independent experiments. X axis: concentration of DAC. Y axis: cell viability after treatment. **B**. Expression of indicated tumor antigens in different human AML cells before and after DAC treatment. Reverse-transcription PCR was performed to check the expression of indicated tumor antigens and the PCR product was run on agarose gel. The result was a representative for at least 3 times independent experiments.

DAC was shown to be capable of increasing the expression of tumor antigens in cancer cells. Here we tested if DAC could affect the expression pattern of tumor antigens in AML cells as well. Mouse AML cell line WEHI3 was used to determine the best concentration of DAC for AML cell treatment. Various concentration of DAC was added to the culture medium of WEHI3 cells for 3 days, and the cell viability was determined. The result shown in **Figure 3A** suggested that the IC50 of DAC in WEHI3 cells was 3.612 µM. We finally choose the concentration of 1 µM of DAC to treat different human AML cell lines to check the effect of DAC treatment on the expression pattern of tumor antigens in AML cells. DAC was added into the culture medium of AML cells for 3 days, then the expression level of different tumor antigens was determined by reverse transcription PCR. As shown in **Figure 3B**, while the expression level of the β-actin, which was used as the internal control, was comparable, the expression level of most of the tested tumor antigens was significantly up-regulated upon DAC treatment in AML cells except K562 cells, which barely responded to the DAC treatment.

### SZU-106-DAC-AML tumor vaccine increased the immunogenicity of tumor vaccine *in vitro*

Next, we tested if a whole-cell tumor vaccine constructed by conjugating SZU-106 to DAC treated tumor cells would result in greater efficacy with respects to the stimulation of host immune response.

**Figure 4:**
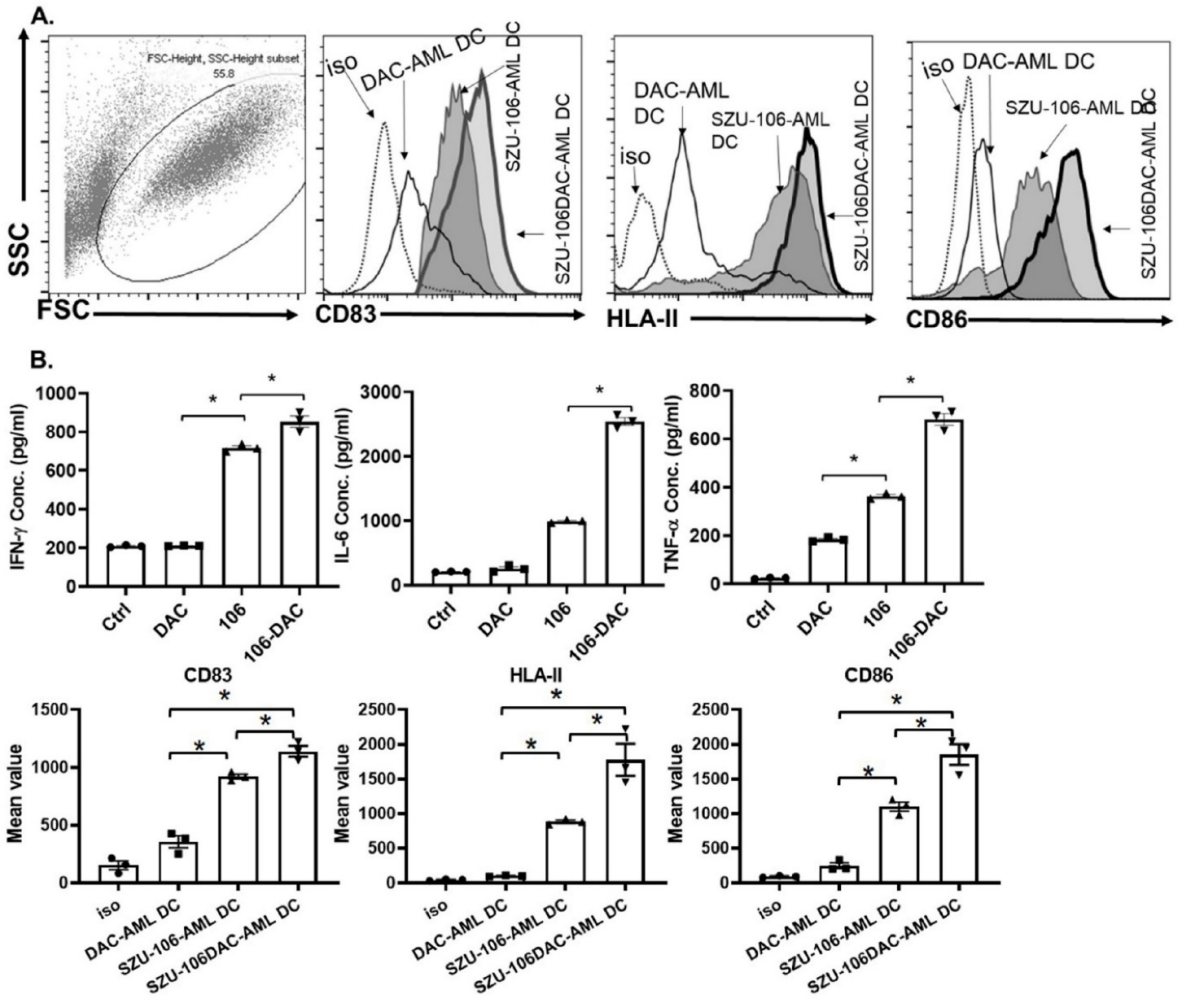
** SZU-106-DAC-AML vaccine enhanced immune response *in vitro.* A.** Isolated human DC cells were treated with indicated tumor vaccines and the phenotype markers for DC activation were measured by flow cytometry. Dotted: isotypes control, thin solid: DAC-AML-DC, thin-shadowed: SZU-106-AML-DC, thick-shadowed: SZU-106-DAC-AML-DC. **B.** Cytokine secretion by isolated T cells upon indicated vaccine treatment. Equal number of T cells were mixed with isolated human DC cells at 10:1 ratio and were treated with indicated tumor vaccines for 24 hours. Cell culture medium was collected to measure the secreted cytokines by T cells upon vaccine treatment. The proinflammatory cytokines, IFN-γ, IL-6 and TNF-α concentration were measured by ELSIA and combined results from three independent experiments, data are mean ± SEM, statistically significant differences were calculated by one-way ANOVA test, *p < 0.05.

U937 cells, a human AML cell line, was chosen to make the whole-cell tumor vaccine. As described in the methods, cells were treated with DAC, conjugated to esterified SZU-106 at 1: 1x10^9^ ratio, and the residual SZU-106 was washes off before use. The resulted whole-cell tumor vaccine was named SZU-106-DAC-AML. Meanwhile, two more whole-cell tumor vaccines, DAC-untreated U937 cells conjugated with SZU-106 and DAC-treated U937 without SZU-106 conjugation, were also constructed to be used as control. DC cells and CD8+ T cells isolated from human peripheral blood were used to test the immunogenicity of the indicated whole-cell tumor vaccines. The FACS result in **Figure 4A** showed the maturation status of DC cells. CD83, HLA-II, and CD86 were used to evaluate the immune response of DC cells upon stimulation. Compared to DAC treated only and SZU-106 conjugated only vaccines, the SZU-106-DAC-AML vaccine demonstrated the strongest potency to promote the expression of CD83, HLA-II, and CD86, suggesting its strongest potential to initiate immune responses compared to the indicated controls.

The abilities of different tumor vaccines to regulate cytokine secretion were also tested in human DC-T cells. CD8+ T cells from human peripheral blood were isolated and cultured. Human DC cells were then co-cultured with CD8+ T cells at 1:20 ratio, and different vaccines, as indicated in **Figure 4B,** were added into the DC-CTL culture media and incubated with cell mixture for 24 hours. Cytokine levels in the culture media were measured after incubation. As shown in **Figure 4B,** the SZU-106-DAC-AML vaccine dramatically increased the ability to stimulate the secretion of IFN-γ, IL-6, and TNF-α compared to the control vaccines in isolated DC-CTL cells.

### SZU-106-DAC-AML inhibited tumor growth and prolonged survival *in vivo*

**Figure 5:**
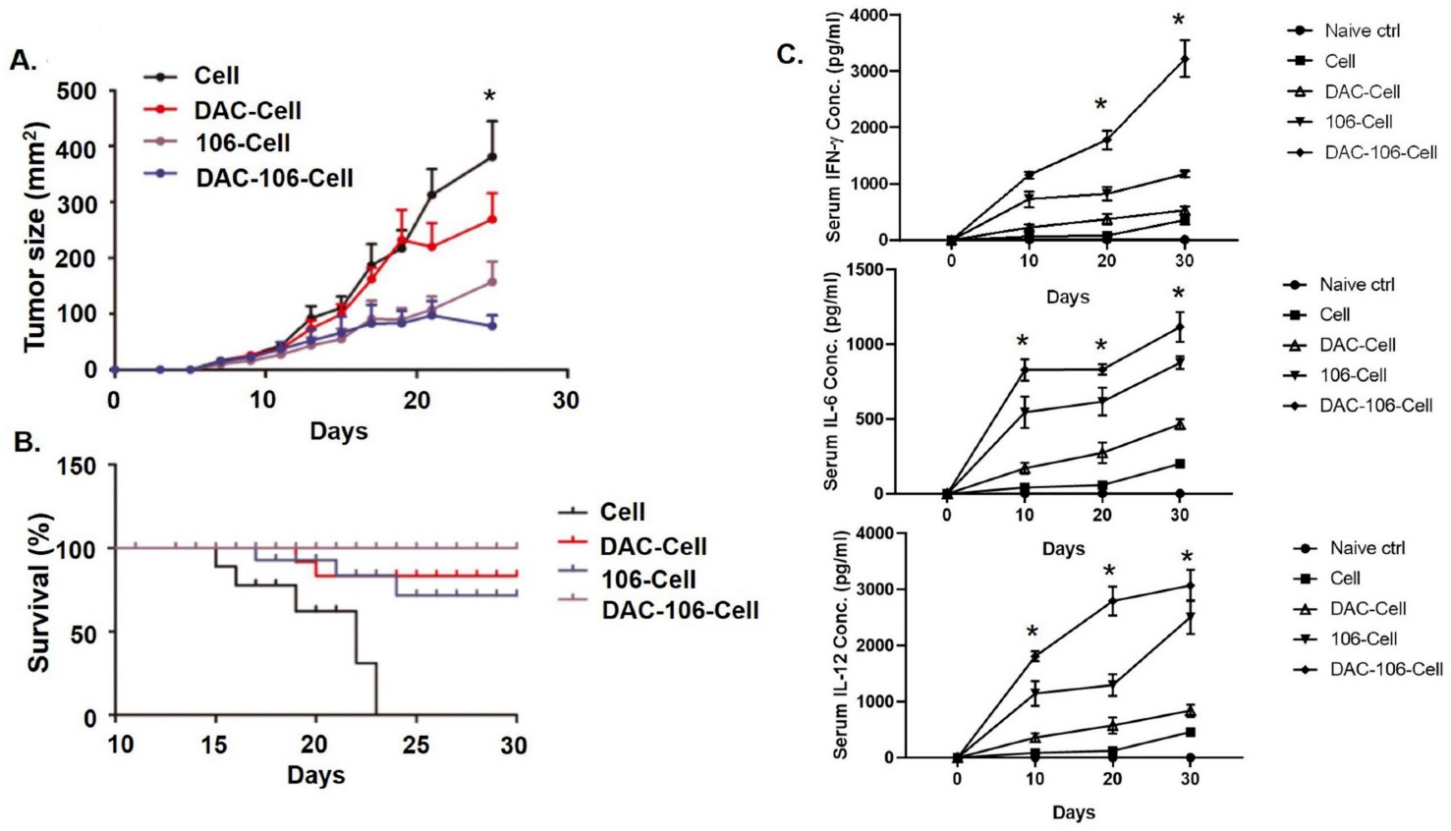
** SZU-106-DAC-AML vaccine inhibited tumor growth and prolonged mice survival. A.** Tumor growth upon indicated tumor vaccine treatment. Mice bearing similar sized tumor were treated with indicated tumor vaccines by subcutaneous injection around the tumors once a week and tumor size was measured every 2 days during treatment. **B.** Mice survival upon indicated tumor vaccine treatment. Mice bearing similar sized tumor were treated with indicated tumor vaccines once a week. Mice survival was monitored. **C.** The peripheral blood cytokines assays showed significantly reduced IFN-γ, IL-6 and IL-12 production in the DAC-106 group compared with others. Comparison of mouse survival, the Kaplan-Meier survival analysis and log-rank test were used, tumor volume and serum cytokines differences were analyzed by using two-way ANOVA test, (n=20 of each experimental group), data are presented as mean ± SEM, *p < 0.05.

Next, we took advantage of the xenograft model to evaluate the effects of the SZU-106-DAC-AML vaccine *in vivo.* U937 cells were inoculated subcutaneously in Balb/c mice. When the tumor diameter reached 5 mm, various whole-cell vaccines were used to treat the tumors by injecting the vaccines subcutaneously around the tumors (n=20 for each group). As shown in **Figure 5A**, the smaller tumor volume in the SZU-106-DAC-AML vaccine treated group compared to that of other groups suggested that tumor growth was significantly inhibited by the SZU-106-DAC-AML vaccine treatment. Remarkably, tumors treated with the SZU-106-DAC-AML vaccine started to shrink after 22 days of treatment **(Figure 5A)**, indicating that the SZU-106-DAC-AML vaccine possesses a promising therapeutic potential.

Mouse survival data were also collected during the xenograft experiment. Results in **Figure 5B** demonstrated that compared to all other control vaccines, the SZU-106-DAC-AML vaccine showed the best ability to prolong the survival of mice bearing AML xenograft tumors. By the end of the experiment, no mice died in the SZU-106-DAC-AML treated group. The peripheral blood cytokines assays showed significantly reduced IFN-γ, IL-6, and IL-12 production **(Figure 5C)** in the DAC-106 group compared with others.

## Discussion

In the present study, we synthesized a novel TLR7 agonist SZU-106 that is suitable to be conjugated to tumor cells. We further investigated the effect of SZU-106 on DC and T cells regarding the initiation of immune responses. It was found that whole-cell tumor vaccine made by conjugating SZU-106 to DAC treated AML cells demonstrated a great potential to exaggerate the immune responses *in vitro* and *in vivo*, indicating its promising therapeutic potential in AML treatment.

The presence of tumor antigens, dendritic cells, and T cells (especially CD8+ T cells) are the three targeted components in the classical antitumor immune response (22). While the efficacy and immunogenicity of the DC vaccines have been demonstrated by numerous clinical studies, the results have been largely misleading. This can be due in part to functional deficiencies in cells comprising traditional vaccine formulations, which are poorly prepared to resolve the immunosuppressive tumor microenvironment restricting DC and cell activity 15, 32. Our new vaccine made by conjugating the TLR7 agonist SZU-106 to DAC treated AML cells were aimed to target all three key components in the classical immune response. DAC treatment increased the expression level of various tumor antigens in cells, the conjugated SZU-106 activated the TLR7 signaling both in DC cells to promote antigen-presenting, and in cytotoxic T cells to promote the secretion of pro-inflammatory cytokines. Taken all together, treatment with our new tumor vaccine improved the host immune responses *in vitro* and* in vivo* **(Figure 4, Figure 5)**.

First, we tried to up-regulate the expression of tumor antigens. It has been reported two decades ago that DNA methylation is the primary silencing mechanism for a set of germ line and tumor-specific genes with a CpG-Rich promoter, for instance, MAGE-A1 33-35. Demethylation agents could restore the expression of MHC class I and its antigen presentation machinery in cancer cells 36-39, and induce the expression of tumor antigens 40, 41, Some studies also revealed that demethylation treatment could up-regulate the expression of costimulatory molecules, such as CD80, CD83, and CD86 38, 42, 43. In our study, DAC was used to treat AML cells to increase the expression of tumor antigens. However, it could not be excluded that DAC treatment could also induce the expression of MHC class I, costimulatory factors, and other unknown molecules to facilitate the exposure of specific tumor antigens to host immune system. Furthermore, DAC is not the only reagent that can modify epigenetics in cancer cells, azacytidine is also reported as a demethylating reagent in a non-random and gene specific manner (34). Testing other demethylating reagents is one of the future directions to explore.

Second, TLRs recognize pathogen-associated molecule patterns and mediate the secretion of necessary cytokines for effective immune response 44. TLR7 is one of the critical TLRs. It is expressed in DC cells and T cells and plays an essential role in immune responses. TLR7 agonists R848 was used clinically as an antiviral and antitumor agent 45. It was also used as an adjuvant for the vaccine 46. The newly synthesized drug SZU-106 demonstrated a comparable potency as R848 to stimulate the TLR7 pathway activation when the concentration was over 10 µM (**Figure 2B**). It is worth noting that when the drug concentration is lower than 1 µM, R848 is better than SZU-106 in terms of TLR7 activation. Modification of SZU-106 that can increase SZU-106 activity as TLR7 agonist may be needed to further improve the immunogenicity of our whole-cell tumor vaccine, while reduce the side effects brought by SZU-106 as much as possible.

Treatment with SZU-106 alone could possibly enhance the host immune response systematically without specific targeting of tumor cells due to the lack of specific antigen. Thus, we conjugated SZU-106 to cancer vaccine cells, hoping that enhancement of tumor antigen exposure and immune responses simultaneously could increase the efficacy of immunotherapy while reduce damages brought by non-specific targeting. The result demonstrated that the SZU-106 conjugated tumor vaccine was more effective, but less toxic (illustrated by improved survival shown in **Figure 4 B**), than both unconjugated vaccine and conjugation only vaccine **(Figure 4 A and B)**, providing a piece of evidence to support the concept that SZU-106 enhanced the host immune response. A possible explanation for the additive effect of the combination of SZU-106 conjugation and DAC treatment is that in addition to activate TLR7 signaling pathways in immune cells, conjugated SZU-106 can also facilitate to initiate tumor antigen specific antigen presentation by bringing more tumor specific antigen to DC cells through binding to the TLR7 molecules on the surface of DC cells.

In the long run, the SZU-106-DAC-AML whole-cell vaccine could also be combined with other treatment strategies such as administration of PD1 monoclonal antibody or PDL1 monoclonal antibody to achieve better treatment outcome. To conclude, our study provides a new alternative immunotherapeutic approach to treat AML.

## Declarations

### Consent to publish and Author Contributions

We confirm that the manuscript has been read and approved by all named authors and that there are no other persons who satisfied the criteria for authorship but are not listed. We further confirm that the order of authors listed in the manuscript has been approved by all of us. G.Z. and G.J. carried out the study. G.Z and L.Y. wrote the manuscript, and G.Z., G.J. W.Z., C.Y., Y.L., J.Z., and L.Z. fabricated the analysis. G.Z and L.Y. helped supervise the project.

### Availability of data and materials

The data that support the findings of this study included in this manuscript, and the original files are available from the corresponding author upon reasonable request.

### Funding Support

This work was supported by the National Natural Science Foundation of China (81170518).

National Science and Technology Major Project, (2018ZX09201003-003).

Shenzhen Science and Technology investigation project (JCYJ20190808123005569).

## Figures and Tables

**Table 1:** Selected names and primers for this study.

	Forward Primer	Reverse Primer
MAGE-A1	CACCTCCTCCTCCTCTCCTC	CACCTCCTCCTCCTCTCCTC
MAGE-A3	GCAAAGCTTCCAGTTCCTTG	AAATGTTGGGTGAGCAGCTT
MAGE-A4	GCAAGTATCGAGCCAAGGAG	TCCCAGATTTCCTCCTCAGA
MAGE-A12	AGAAGTGGACCCCTTTGTCC	GGATCACTATTGGGCACCTG
MAGE-B1	TGCTGCAGCTGTGTCATGTA	TGGCCACTAGGGTTGTCTTC
MAGE-B2	CTTCAAGCTCTCCTGCTGCT	GGAAGTGCTCCCTGAACCTT
MAGE-E1	AGAGCATCACAGCCCTCATT	TCAGGTGGATCCCAAACTTC
PRAME	CTGTACTCATTTCCAGAGCCAGA	TATTGAGAGGGTTTCCAAGGGGTT
SSX-1	CTAAAGCATCAGAGAAGAGAAGC	AGATCTCTTATTAATCTTCTCAGAAA
SSX-2	GGTGCTCAAATACCAGAGAAG	GGTGCTCAAATACCAGAGAAG
SSX-4	GTTCTCAAATACCAGAGAAGATG	CTCTGCTGGCTTCTCGGGCG
GAGE-2	GCCTAGACCAAGACGCTACG	CCTTCTTCAGGCGTTTTCAC
GAGE-4	GCCTAGACCAAGGCGCTAT	CCTTCTTCAGGCGTTTTCAC
GAGE-7	GGAATTCATGAGTTGGCGAGGAAGATCGACC	CCGCTCGAGTTAACACTGTGATTGCTTTTCACC
XAGE-1	GTATCCGAGTCCCAGAAGCA	GATTTATCCCCGGTGTTTGA
NY-SAR-35	AAAGCGAAGGGGAGGAATAG	GGGCAGGATATGTCCATTTG
NY-ESO-1	GCTTCAGGGCTGAATGGAT	AAAAACACGGGCAGAAAGC
MAD-CT1	AGGTGTACAGGCAGCAGTTG	TCTGCATGTTCTCTTCCTGGT
TPX-1	AGAGGACCGCAAAACCAGTA	TTCCTTGTTGGTACGGGGTA
NXF-2	TGAAACCCTGCAAGGAAAAC	GCACTGAGGGAGTCCACAAT
β-actin	TCTGGCACCACACCTTCTACAATGAGCTGCG	CGTCATACTCCTGCTGATCCACATCTC
